# The influence of management of tooth wear on oral health-related quality of life

**DOI:** 10.1007/s00784-018-2355-8

**Published:** 2018-02-03

**Authors:** Bernadette A. M. M. Sterenborg, Ewald M. Bronkhorst, Peter Wetselaar, Frank Lobbezoo, Bas A. C. Loomans, Marie-Charlotte D. N. J. M. Huysmans

**Affiliations:** 1 Radboud Institute for Health Sciences, Department of Preventive and Restorative Dentistry, Radboud University Medical Center, P.O. Box 9101, NL, 6500 HB Nijmegen, The Netherlands; 2 Department of Oral Kinesiology, Academic Centre for Dentistry Amsterdam (ACTA), University of Amsterdam, Amsterdam, The Netherlands

**Keywords:** Oral health-related quality of life, Tooth wear, Mouth rehabilitation, OHIP-49, OES, Management

## Abstract

**Objective:**

The purpose of this study was to identify the level of oral health-related quality of life and orofacial appearance in patients with moderate to severe tooth wear. Patients with and without a request for restorative treatment were included.

**Methods:**

One hundred twenty-four patients (98 men, 26 women, mean age: 40.5 ± 8.8 years) with moderate to severe tooth wear were included. Patients without a request for help received a non-restorative treatment of counseling and monitoring. Patients with a request for restorative treatment were treated with a full rehabilitation using composite resin restorations. Oral Health Impact Profile (OHIP-NL) and Orofacial Esthetic Scale (OES-NL) questionnaires were filled in at baseline and after 1 year.

**Results:**

Counseling and monitoring group: baseline OHIP-NL score was 0.4 ± 0.3, baseline summary score of OES-NL was 48 ± 7.0, and baseline impression score was 7.1 ± 1.2. Scores had not changed significantly after 1 year (*p =* 1.00 after Bonferroni correction).Restoration group: baseline OHIP-NL score was 0.8 ± 0.6, baseline summary score of OES-NL was 38 ± 10, and baseline impression score was 5.9 ± 1.5. Scores had improved significantly after 1 year (*p* < 0.001 after Bonferroni correction).

**Conclusions:**

Counseling and monitoring did not result in a significant deterioration and restorative treatment resulted in a significant improvement of oral health-related quality of life (OHRQoL) and orofacial appearance in this patient group.

**Clinical significance:**

In patients with moderate to severe tooth wear, without functional and esthetical problems, counseling and monitoring may be an appropriate treatment option. Restorative treatment in patients with a need for treatment results in an improved OHRQoL. OHIP and OES questionnaires may be used to monitor changes in clinically relevant symptoms.

## Introduction

Tooth wear is a loss of dental hard tissues which is irreversible, multifactorial, non-carious, and may, in some situations, lead to a pathological situation with functional or esthetical problems [19]. It is known that tooth wear may have an impact on patients’ satisfaction with their dentition regardless of tooth wear severity or personal factors. Mostly, patients complain about tooth sensitivity (dentin exposure), dental pain (involvement of the pulp), poor esthetics (shortened clinical crown length), and functional impairment (difficulties with chewing due to occlusal alterations and dental tissue loss) [25]. Dissatisfaction with their appearance is the most common complaint [7, 42], and problems with appearance and function motivate patients to seek treatment [15].

Dental disease may influence an individual’s capacity to live comfortably, be successful in employment, enjoy life, experience relationships, and possess a positive self-image [39]. Even though dental disease is rarely life-threatening, it can still affect quality of life [23, 27]. Different levels of oral status have various impacts on daily living, and therefore, both the clinical status and psychological dimensions should be addressed whenever dental treatment needs are being assessed [9, 23].

In general, three approaches are used to measure oral health-related quality of life (OHRQoL). These are the following: social indicators, global self-ratings, and multiple-item questionnaires. This conceptual model is based on the World Health Organization (WHO) document “the WHO international classification of impairments, disabilities and handicaps” [26]. The Oral Health Impact Profile (OHIP) is the most frequently used oral-specific measure for oral health-related quality of life. It is a questionnaire that contains 49 statements organized in seven domains: functional limitation, physical pain, psychological discomfort, physical disability, psychological disability, social disability, and handicap [36]. The validity and reliability of the original English version of the OHIP have been evaluated in several epidemiological and cross-cultural studies [2, 3, 17, 36]. A Dutch translation of the OHIP has also been validated [40].

For the specific problems regarding appearance, a separate questionnaire has been developed: the Orofacial Esthetic Scale (OES), which aims to obtain a characterization of the orofacial esthetics [21, 22]. It consists of an eight-item instrument, which may be used to assess how patients perceive their dental and facial esthetics. The OES has also been validated for the Dutch language and showed good psychometric properties in a population with self-reported tooth wear [43].

Patients with severe tooth wear were shown to have an impaired OHRQoL, comparable to that of edentulous patients [30]. A recent study in the UK explored the association between tooth wear and quality of life among adults in the UK, independently of socio-demographic factors and other common oral conditions. It concluded that severe tooth wear was associated with psychological impacts on people’s life [24]. On the other hand, a study in 2011 in a sample of university students showed that tooth surface loss into dentine was prevalent among young adults, but that it had little impact on OHRQoL [10].

The purpose of this study was to identify the level of OHRQoL and orofacial appearance in patients with moderate to severe tooth wear. Patients were grouped into those with or without a request for restorative treatment. At baseline and after 1 year, the OHIP-NL and the OES-NL were completed, to analyze OHRQoL before and after non-restorative or restorative treatment.

## Material and methods

Patients with tooth wear were referred by general dental practitioners to the Department of Dentistry of the Radboud University Medical Center (Nijmegen, The Netherlands). The inclusion took place in the period September 2011 until June 2014. Ethical approval (for a larger study of which the current study is a part) was sought and granted before the study was undertaken (ABR code: NL31371.091.10). All patients who were asked to participate agreed and signed an informed consent document before entering the study.

### Inclusion/exclusion criteria

The following inclusion criteria were used for selection of the patients: (1) ≥ 18 years;

(2) moderate/severe tooth wear (TWI ≥ 2); (3) full dental arches, with a maximum of one missing tooth in the posterior area; (4) absence of serious general health problems (ASA score ≤ 3).

The following exclusion criteria were used: (1) mouth opening < 3.5 cm, (2) temporomandibular pain or dysfunction, (3) periodontitis (pockets > 4 mm), (5) active dental caries or endodontic problems.

### Treatment

On the basis of the severity of the tooth wear and their objective and subjective treatment need, the patients were treated either with counseling and monitoring or with restorations.

Counseling and monitoring: This group contained patients without a clear request for restorative treatment. Counseling included information about the main cause of their tooth wear (chemical and/or mechanical). If necessary, specific preventive measures were advised (e.g., consultation with family doctor in case of gastroesophageal reflux disease, dietary advice, or the fabrication of a hard occlusal stabilization splint for nighttime usage).

Restorative treatment: This group contained patients with a clear request for restorative intervention due to functional (difficulties with chewing, discomfort) or esthetic problems. These patients were treated restoratively with a full rehabilitation using composite resin restorations, including an increase of vertical dimension of occlusion [29].

As these groups were obviously different on an important aspect, namely request for treatment, no treatment outcome comparisons between the groups were made.

### Assessment of tooth wear

In order to score tooth wear, diagnostic study models were assessed. The tooth wear index (TWI) of Smith and Knight was used [37], with scores ranging between 0 (no loss of enamel surface characteristics) and 4 (complete enamel loss, pulp exposure, or secondary dentin exposure). Every surface of each tooth was assessed and the highest score was recorded. The highest score in the whole mouth determined the patient TWI score. Repeated measurements were made after 2 weeks on a random sample of 15 patients to determine reliability.

### Questionnaires

At baseline and after 1 year, patients were asked to complete the 49-item Oral Health Impact Profile-NL questionnaire and Orofacial Esthetic Scale-NL questionnaire [40, 43]. Following explanation of the questionnaire by a research assistant, patients completed the questionnaire without assistance. For each statement in the OHIP-NL, patients were asked to score how frequently they experienced the impact of this statement during the last month because of problems with their teeth, mouth, or dentures. The answers were scores on 5-point ordinal scales, ranging from never (0), hardly ever (1), occasionally (2) and fairly often (3), to very often (4) [40]. Higher scores imply a more impaired OHRQoL. Three questions refer exclusively to dentures (question nos. 9, 18, and 30) and were therefore excluded from our analysis. The sensitivity of the OHIP to change has been investigated and it was concluded that summing up scores was a good method to detect changes [1]. In our analysis, we used the summary score as suggested by John et al. [18].

In the OHIP questionnaire are questions included about the orofacial appearance but to get a better understanding of the impact of the orofacial appearance, it was decided to add the OES-NL questionnaire. The OES-NL consisted of eight questions about the appearance of the face, profile, mouth, tooth alignment, tooth shape, tooth color, and gums. The last question is an overall impression question and can be analyzed separately. The answers are scores on 11-point ordinal scales, ranging from very dissatisfied (0) to very satisfied (10). Lower scores imply more impaired orofacial appearance. For the summary score, items 1 to 7 are used; for the overall impression score, item 8 is used.

### Missing data

If there were more than five questions on the total OHIP-NL or more than two questions from within one of the seven domains unanswered, the data was discarded. For the remainder, missing responses to individual questions were replaced with the mean value of the coded response of the corresponding question [34]. If there was more than one answer missing on the total OES-NL, the questionnaire was discarded.

### Statistical analysis

Reliability for the TWI scores was calculated using a weighted Cohen’s kappa.

Data of the questionnaires at baseline was compared with data after 1 year using paired *t* tests, *p* < 0.05. Changes in summary score were analyzed for each of the two groups separately. Correction for multiple testing for the OHIP-NL outcome is mandatory and therefore the Bonferroni test was used. Eight tests were done, so the *p* values were multiplied by 8.

For the OES-NL, summary score (questions 1 to 7) and the overall impression score (question 8) were analyzed separately with paired *t* tests (95% CI, *p* < 0.05). In the OES-NL questionnaire, two questions refer specifically to the shape and color of teeth: questions 5 and 6. These outcomes and their correlation with the specific questions about esthetics in the OHIP-NL (3, 22, 31) were analyzed using Spearman’s rank correlation. All statistical analyses were performed with the Statistical Package for Social Sciences (SPSS 22).

## Results

In total, 124 patients (98 men, 26 women, mean age: 40.5 ± 8.8 years) participated in this study. The counseling & monitoring group consisted of 46 patients (37 men and 9 women, mean age: 40.8 ± 9.3 years), with a mean maximum TWI score of 2.9 ± 0.5. The restoration group consisted of 78 patients (61 men and 17 women, mean age: 40.3 ± 8.5 years), with a mean maximum TWI score of 3.3 ± 0.4. The TWI score had a weighted Cohen’s kappa score of 0.60. None of the OHIP-NL questionnaires had to be discarded for missing answers so there was no need for data imputation. For the OES-NL, 15 questionnaires had to be discarded due to missing answers. None of the patients dropped out of this study.

**Fig. 1:**
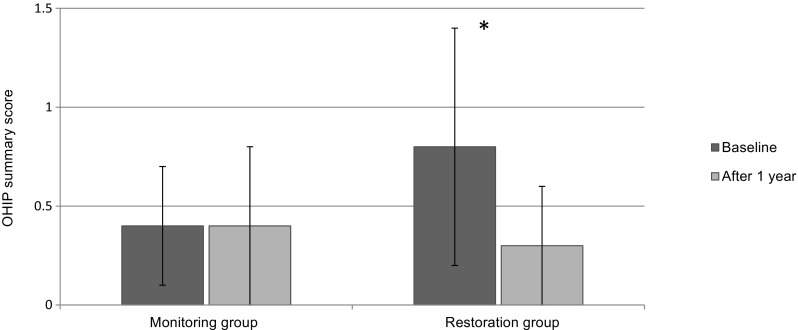
The summary scores of the OHIP-NL (mean (± SD)) within the counseling and monitoring group and restoration group at baseline and after 1 year. *p < 0.001

The results for the OHIP-NL are visualized in Fig. 1 and the analysis is presented in Table 1. In the counseling & monitoring group, the OHIP-NL showed no change over a period of 1 year: with a mean overall change over 1 year of 0.01 (SD 0.3; *p* = 1.0). In the restoration group, a statistically significant impact on OHRQoL was found: mean overall change over 1 year of − 0.5 (SD 0.5; *p* < 0.001).

**Table 1:** Comparison (*t* test with Bonferroni correction included) of the counseling & monitoring group and the restoration group Baseline and after 1 year for the OHIP summary score (± SD)

Variable	Mean			95% CI of diff		
	Baseline	After 1 year	Mean difference	*P* value	Lower	Upper
Summary score counseling & monitoring group	0.4 (0.3)	0.4 (0.4)	0.01 (0.3)	1.0	− 0.07	0.09
Summaryscore restoration group	0.8 (0.6)	0.3 (0.3)	− 0.5 (0.5)	< 0.001	− 0.6	0.4

**Fig. 2:**
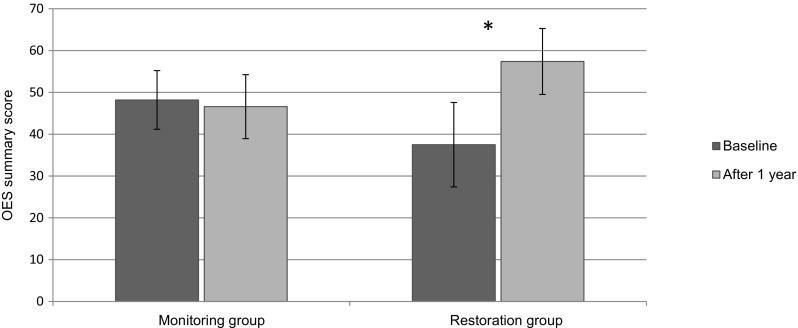
The summary scores of the OES-NL (mean (± SD)) within the counseling and monitoring group and restoration group at baseline and after 1 year. *p < 0.001

The results for the OES-NL are visualized in Fig. 2, showing a similar effect as seen for the OHIP, and the analysis is presented in Table 2. Both summary score (average change − 2, *p* = 0.12) and overall impression score (average change − 0.1; *p* = 0.71) did not change significantly in the counseling & monitoring group. Patients in the restoration group did show significant changes for both the summary score (average change 19; *p* < 0.001) and overall impression score (average change 2.5; *p* < 0.001).

**Table 2:** Comparisons (*t* test) between baseline and after 1 year for the counseling & monitoring group and the restoration group, using the Orofacial Esthetic Scale summary score and the overall impression score (± SD)

	*N*	Mean baseline	Mean after 1 year	Mean difference	95% CI of diff	*P* value
OES summary score						
Counseling & monitoring group	44	48 (7.0)	46 (7.6)	− 2 (6.7)	− 3.6… 0.4	0.12
Restoration group	65	38 (10)	57 (7.9)	19 (14)	16 … 23	< 0.001
OES overall impression score						
Counseling & monitoring group	44	7.1 (1.2)	7.0 (1.1)	− 0.1(0.9)	− 0.44… 0.29	0.71
Restoration group	65	5.9 (1.5)	8.4 (1.1)	2.5 (2.0)	2.0… 3.0	< 0.001

In the counseling & monitoring group, there was no change for questions 5 (− 0.27 ± 1.5 (*p* = 0.26)) and 6 (− 0.34 ± 1.4 (*p* = 0.13)). In the restoration group, however, a significant increase was observed for both questions: 4.2 ± 2.8 (Q5, *p* < 0.001) and 3.2 ± 2.4 (Q6, *p* < 0.001). For OHIP-NL questions 3, 22, and 31, no change occurred for the counseling and monitoring group (*p* ≥ 0.15), while all questions showed change for the restoration group (*p <* 0.001). These groups of questions from the two questionnaires showed no correlation for the counseling & monitoring group (Spearman’s Rho baseline − 0.25, *p* = 0.10, 1 year 0.021, *p* = 0.89), but a significant positive (considering the opposite scales) correlation for the restoration group (baseline − 0.64, *p* < 0.001; 1 year − 0.32, *p* < 0.001).

## Discussion

The purpose of this study was to investigate the impact of moderate to severe tooth wear on the oral health-related quality of life, and the effect 1 year after treatment either with counseling and monitoring or with composite restorations. To understand the impact of the esthetics on orofacial appearance better, the OES-NL was included in addition on the OHIP-NL. We did not aim to compare the two treatment modalities, as they are not equal alternatives, but each indicated in a different clinical situation. It was observed that patients with roughly comparable objective degrees of tooth wear (TWI 2.9 vs 3.3) reported a very different impact of this wear on their OHRQoL (OHIP-NL 0.4 vs 0.8). A major improvement in oral health-related quality of life (OHRQoL) resulted after restorative treatment, and patients who received counseling and monitoring only showed a stable OHRQoL over a period of 1 year.

Where a functional or esthetical demand for treatment is present, a dynamic treatment concept based on minimally invasive principles is generally preferred. In this concept, the word “dynamic” means retaining options for a new or repeated treatment when the first treatment fails [8]. Minimally invasive means that restorations are placed with a minimum of additional tooth loss or iatrogenic damage. In total rehabilitations of severely worn dentitions, direct composite resin restorations have been shown to be successful [4, 14, 28, 31]. Even indirect composite resin restorations can be used in a minimally invasive way. The results of these treatment options seem to be very satisfying; however, no data is presented about the effect of such treatment on OHRQoL. As far as we know, this is the first report showing that a restorative treatment resulted in a statistically significant improvement of OHRQoL in patients with tooth wear. And perhaps equally important, we showed that in a group of patients with considerable tooth wear who received only counseling treatment, an option which may truly be called minimally invasive, no deterioration of OHRQoL could be observed over a period of 1 year.

With 97% male patients, the gender imbalance in this study is notable. A slight disbalance was also observed in studies with adolescents, in which it was found that more boys than girls had erosive tooth wear [12, 41]. In adult subjects with extensive (> 10% of surfaces) levels of tooth wear, highly significantly more men than women were affected [38]. Despite the limited data available on tooth wear rates, tooth wear progression rates were reported to be higher in males in some studies [11, 13], although other reports failed to find a difference [16, 20, 33]. The explanation for this higher prevalence of tooth wear in males is not yet available. Wetselaar et al. suggest that differences in diet may be a reason, due to the evidence that male adolescents and adults consume more acidic drinks than women, leading to possible more (erosive) tooth wear [46]. They also suggest that men’s masticatory muscles exert higher forces, which may cause more (mechanical) tooth wear.

Another interesting aspect is the relatively young age of the patients in the present study, with a mean age of 40.5 ± 8.8 years. Although tooth wear is partly an age-dependent physiological phenomenon, at every age there may be individuals showing a pathological degree or rate of wear. It may be argued that the severity of tooth wear should be viewed relative to the “normal” wear of the age group, and that the occurrence of severe wear is thus independent of age [6]. Other studies reporting similar proportions of severe levels of wear in different age groups support this theory [5, 11, 38, 45].

The higher degree of impact on OHRQoL of tooth wear in the group who received restorative treatment could be explained by both reduced self-confidence due to a compromised appearance and by pain and sensitivity, causing more effect on their OHRQoL. Patients with pain have been shown to have higher OHIP scores compared to patients without pain [30, 32, 35]. Whereas patients with tooth wear had similar OHIP scores to edentulous individuals, scores from patients with painful temporomandibular disorders were even higher, indicating that pain is an important aspect of OHRQoL [30]. Wetselaar et al. described reasons for a tooth wear patient to seek help: sensitivity and/or pain, difficulties with chewing/eating, impaired orofacial esthetics, and crumbling of dental hard tissue and/or dental restorations [44]. Patients who received a restorative treatment experienced the ability to eat and drink without pain after the treatment resulting in a major improvement in their OHRQoL. Li et al. showed that dentists, when making treatment decisions, should consider not only the patients’ objective clinical characteristics but also the impact of the condition on quality of life [24]. The decision for restorative treatment in the present patient group was strongly influenced by their subjective complaints of impaired appearance and pain.

Impaired orofacial esthetics was the highest scoring complaint in the restoration group. In a previous study in the Netherlands, the Dutch version of the OES was used for patients with and without self-reported tooth wear [43]. Summary scores were 46.4 ± 12.1 and overall impression scores 6.9 ± 1.7 for patients with tooth wear (*n* = 307) and 49.7 ± 11.3 and 7.2 ± 1.6, respectively, for patients without tooth wear (*n* = 276). These results are comparable to those of the present study for the counseling and monitoring group. The restoration group reported an improvement of their orofacial appearance using the OES-NL. For the counseling and monitoring group, there was no significant change after 1 year. In an ongoing study, the satisfaction with the facial appearance over a longer period of time will be investigated, to evaluate the long-term effects.

We observed no significant correlation between the esthetics-related questions of the OES-NL and the OHIP-NL for the counseling and monitoring group. For the restoration group, however, we observed significant correlations ranging from − 0.32 to − 0.64. This may be explained by the higher levels of impact of the esthetic aspect in the restoration group.

## Conclusion

It was shown that in patients with moderate to severe tooth wear, but without a request for restorative treatment, no changes were observed in the level of their oral health-related quality of life and orofacial appearance over a period of 1 year. Patients who asked for restorative treatment, often for reasons of esthetics or pain, experienced a significant improvement of their oral health-related quality of life and orofacial appearance after restorative treatment.
